# Supplementary material for: Universal third-trimester ultrasonic screening using fetal macrosomia in the prediction of adverse perinatal outcome: A systematic review and meta-analysis of diagnostic test accuracy
Source: PLoS Med. 2020 Oct 13;17(10):e1003190. doi: 10.1371/journal.pmed.1003190 (PMC7553291; doi:10.1371/journal.pmed.1003190)
Supplement: S1 Text — (DOCX) [file pmed.1003190.s002.docx]

**S1 Text: Literature search strategy for Medline and Embase (from inception to May 2020)**

1. exp fetus echography/

2. ultrasonography, prenatal.mp.

3. exp ultrasound/

4. ultraso*.mp.

5. sonograph*.mp.

6. exp biometry/

7. USS.mp.

8. estimated fetal weight.mp.

9. EFW.mp.

10. abdominal circumference.mp.

11. AC.mp.

12. exp macrosomia/

13. macrosomi*.mp.

14. exp fetus weight/

15. fetal weight.mp.

16. exp birth weight/

17. birthweight.mp.

18. large for gestational age.mp.

19. LGA.mp.

20. large fetus.mp.

21. exp brachial plexus injury/ or brachial plexus injury.mp.

22. exp shoulder dystocia/ or shoulder dystocia.mp.

23. 1 or 2 or 3 or 4 or 5 or 6 or 7 or 8 or 9 or 10 or 11

24. 12 or 13 or 14 or 15 or 16 or 17 or 18 or 19 or 20 or 21 or 22

25. 23 and 24

26. exp pregnancy/

27. 25 and 26
