# Supplementary material for: Universal third-trimester ultrasonic screening using fetal macrosomia in the prediction of adverse perinatal outcome: A systematic review and meta-analysis of diagnostic test accuracy
Source: PLoS Med. 2020 Oct 13;17(10):e1003190. doi: 10.1371/journal.pmed.1003190 (PMC7553291; doi:10.1371/journal.pmed.1003190)
Supplement: S1 Table — (DOCX) [file pmed.1003190.s003.docx]

S1_Table. Characteristics of the studies included in the meta-analysis

| **First Author (Year)** | **Type of Study, Setting** | **Total number of fetuses (number of LGA fetuses at birth), risk, and selection**  **(All singleton, non anomalous unless otherwise stated)** | **Index test**  **(Blinding)** | **Gestational age at ultrasound** | **Reference standard** | **Gestational age at delivery** | **Other comments**  **(Inclusion of T1DM, T2DM and GDM)** |
| --- | --- | --- | --- | --- | --- | --- | --- |
| **Aviram 2017** | Retrospective cohort,  Single Hospital,  Israel | N= 7996 (1618)  Risk: Mixed  Selection: Mixed risk, term only. Excluded SGA deliveries, intrapartum and SROM. | EFW (20 formulas)  Hadlock (AC/FL/BPD)  Hadlock (AC/FL/HC)  Hadlock (AC/FL/BPD/HC)  Hadlock (AC/FL)  Hadlock (AC/BPD)  Shepard (AC/BPD)  Threshold: >90^th^ centile  Blinded: No | Within 1 week from delivery. | BW >90^th^ centile | Mean for LGA group: 39.4 weeks, mean for AGA group: 38.3 weeks | DM/GDM: Included (21% for LGA, 14% for AGA) |
| **Balsyte 2009** | Retrospective cohort,  Single Hospital, Switzerland | N= 1062 (135)  Risk: Mixed  Selection: Term only. | EFW  Hadlock (AC/FL/HC)  Threshold: >4000g  Blinded: No | Within 1 week from delivery. | BW >4000g | Mean 39.3 weeks. | DM/GDM: Not reported |
| **Benecerraf 1988** | Retrospective cohort,  Single hospital, Boston, MA, USA | N= 1301 (324)  Risk: Mixed  Selection: Included all pregnancies apart from breech and multiples. | EFW (Birnholz)  Threshold: Threshold: >4000g, >3800g  Blinded: No | Within 1 week from delivery. | BW >4000g | Not specified | DM/GDM: Included |
| **Ben-Haroush 2007** | Prospective cohort,  Single Hospital,  Israel | N= 259 (23)  Risk: Universal  Selection: Routine scan. Included SGA. Excluded hypertensives and diabetics. | EFW  Hadlock (AC/FL/BPD)  Threshold: >90^th^ centile  Blinded: No | Mean 32 weeks | BW >4000g | Mean 39 weeks. | DM/GDM: Excluded |
| **Ben-Haroush 2008** | Retrospective cohort,  Single Hospital,  Israel | N= 1925 (140)  Risk: Mixed  Selection: Term only. | EFW  Hadlock (AC/FL)  EFW + AFI  Threshold: EFW >4000g, AFI >95mm (60^th^ centile)  Blinded: No | Interval from USS to delivery 2.5 days | BW >4000g | Mean for LGA 40 weeks,  Mean for normal BW 39.4 weeks | DM/GDM: Excluded |
| **Benson 1991** | Retrospective cohort, Boston, MA, USA | N= 412 (32)  Risk: Mixed  Selection: Not specified. Excluded diabetics. | EFW  Hadlock (AC/FL/BPD)  Threshold: >90^th^ centile  Blinded: No | Within 1 week from delivery | BW> 90^th^ centile | Not specified | DM/GDM: Excluded |
| **Burkhardt 2014** | Retrospective cohort  Single Hospital, Zurich, Switzerland | N= 12,794  Risk: Mixed  Selection: All term, with vertex presentation with scan with 7days | EFW, AC  Hadlock (AC/FL/BPD)  Threshold:  >4000g, >4500g  >35cm, >39cm  Blinded: No | Within 1 week from delivery | Shoulder dystocia | 281 days fro SD  278 days for no SD | DM/GDM: 7.5% for those with SD  2.7% for those without SD. |
| **Chauhan 2006** | Retrospective cohort  Single Hospital, Houston, TX, USA | N= 1954 (119)  Risk: Mixed  Selection: Pregnancies undergoing fetal surveillance. Included SGA, hypertensives (22%) and SROM (5%). | EFW  Hadlock (AC/FL/BPD)  Threshold: >90^th^ centile  Blinded: No | Within 4 weeks from delivery. 64% within 7 days from delivery. | BW >90^th^ centile | 34% preterm | DM/GDM: Included (13%) |
| **Chervenak 1989** | Prospective cohort  Single Hospital, New Jersey, USA | N= 317 (81)  Risk: Low  Selection: Uncomplicated pregnancies after 41 weeks’ gestation. | EFW  Hadlock AC/BPD or AC/FL if BPD not available  Threshold: >4000g  Blinded: Not clear | >41 weeks | BW >4000g | Mean 42 +/- 0.6 weeks | DM/GDM: Excluded |
| **Cohen 2010** | Retrospective cohort  Single Hospital, Montreal, Canada | N= 1099 (105)  Risk: Mixed  Selection: Only included pregnancies with USS on the same or next day as delivery | EFW  Hadlock (AC/FL/BPD/HC)  Threshold: >90^th^ centile  Blinded: No | On the same or next day of delivery. | BW >4000g | Mean 275.2 days. | DM/GDM: Included (11.6%) |
| **Crimmins 2018** | Retrospective cohort  Single hospital, Baltimore, Maryland, USA | N= 945 (40)  Risk: Mixed  Selection: All pregnancies >34 weeks gestation with normal oGCT. | AFG defined as EFW >90^th^ centile (Hadlock- AC/FL/BPD) or AC >95th centile.  Polyhydramnios >25cm  Threshold: As above.  Blinded: No | >34 weeks | BW >4000g  Shoulder dystocia  NICU admission | Not specified. | DM/GDM: Excluded |
| **Cromi 2007** | Retrospective cohort,  2 hospitals, Swtzerland | N= 1026 (53)  Risk: Mixed  Selection: All singletons >34 weeks gestation with USS within 4 weeks of delivery. Excluded SROM. | EFW, AC  Hadlock (AC/FL/BPD)  Threshold: >95^th^ centile  Blinded: No | Within 4 weeks of delivery.  Mean 37.3 weeks | BW >4000g  BW>4500g | >34 weeks  Mean 39.2 weeks | DM/GDM: Included (8.8%) |
| **De Reu 2008** | Retrospective cohort,  Single Hospital, Netherlands | N= 3449 (285)  Risk: Universal  Selection: Women with no risk factors or pathology. Did not exclude SGA. | AC  Threshold: >75^th^ /90^th^/95^th^ centile  Blinded: No | Between 27 and 33 weeks. | BW >90^th^ centile,  BW >95^th^ centile | Mean 278.7 days | DM/GDM: Excluded |
| **Freire 2010**  **(Portuguese)** | Retrospective cohort, 2 hospitals, Brazil | N= 114 (8)  Risk: Mixed  Selection: Those with USS within 7 days of delivery | EFW  Hadlock (AC/FL/BPD/HC)  Threshold: >90^th^ centile  Blinded: No | Within 7 days of delivery | BW >90^th^ centile | 15.6% preterm,  84.4% at term | DM/GDM: Not reported |
| **Galvin 2017 (GENESIS study)**  **(Abstract)** | Prospective cohort  Large multi-centre study, Ireland | N= 2336 (not known)  Risk: Low  Selection: Term, uncomplicated, cephalic only. | EFW (Not specified)  Threshold: 4000g  Blinded: Yes | Between 39+0 and 40+6 weeks | Shoulder dystocia  NICU admission | Not specified. | DM/GDM: excluded |
| **Gilby 2000** | Retrospective cohort,  Single Hospital, Florida, USA | N= 1996 (318)  Risk: Mixed  Selection: All singleton >36 weeks with USS within 1 week from delivery. | AC  Threshold: >35cm, >38cm  Blinded: No | Within 1 week from delivery | BW >4500g | >36 weeks  Mean not reported. | DM/GDM: Not reported |
| **Hasenoehrl 2006** | Prospective cohort, Single hospital, Austria | N= 200 (33)  Risk: Low  Selection: Included those with USS within 1 week. Excluded only fetal anomaly. | EFW (Schild)  Threshold: >4000g  Blinded: No | Mean 39.2 weeks | BW >4000g | Mean interval 2.0 days. | DM/GDM: Not reported |
| **Hendrix 2000** | Prospective (RCT)  Georgia, USA | N= 367 (39)  Risk: Low  Selection: Term only. | EFW  Hadlock AC/BPD  Threshold: >4000g  Blinded: No | >37 weeks | BW >4000g | Mean 39.1 weeks | DM/GDM: Not reported |
| **Henricks 2003** | Prospective cohort,  South Carolina, USA | N= 256 (21)  Risk: Universal  Selection: Term only. | AC  Threshold: >35cm  Blinded: No | >37 weeks | BW >4000g | Mean 39.1 weeks | DM/GDM: Not reported |
| **Humphries 2002** | Retrospective cohort,  South Carolina, USA | N= 238 (29)  Risk: Mixed  Selection: Term only, with USS within 2 weeks. | EFW  Combs (AC/FL/FL)  Threshold: >4000g  Blinded: No | Within 2 weeks of delivery | BW >4000g | >37 weeks | DM/GDM: Not reported |
| **Kayem 2009** | Prospective cohort,  Multiple hospitals, France and Belgium | N= 1689 (124)  Risk: Low  Selection: As part of a prospective cohort for breech. Term only, with USS within 10 days of delivery. | AC  Threshold: >36.3cm  Blinded: No | Within 10 days of delivery. | BW >4000g | Median 39 weeks | DM/GDM: Not reported |
| **Kehl 2011** | Prospective cohort, Single Hospiotal, Germany | N= 258 (30)  Risk: Universal  Selection: Term only with vertex presentation and USS within 3 days of delivery. | AC  Threshold: >36cm  Blinded: No | Within 3 days of delivery | BW >4000g | 40+5 weeks for AC>36cm  39+6 weeks for AC <36cm | DM/GDM: Not reported |
| **Khan 2019** | Retrospective cohort,  2 HNS Hospitals, London, UK | N= 45847 (4229)  Risk: Universal  Selection: Term only. | EFW  Hadlock (AC/FL/HC)  Threshold: >90^th^ centile  Blinded: No | Between 35+0 and 36+6 weeks  Mean 36.1 weeks | BW >90^th^ centile  BW >97^th^ centile | Mean 39.9 weeks | DM/GDM: T1DM/T2DM Included (0.7% for non-LGA, 2.1% for LGA) |
| **Levine 1992** | Retrospective cohort,  Single Hospital, New York, USA | N= 406 (68)  Risk: Mixed  Selection: Term only. Included pregancies with diabetes (22%) and previous CS (20%) | EFW  Hadlock (AC/FL/HC)  Threshold: >90^th^ centile  Blinded: No | 5-10 days before delivery | BW >90^th^ centile | Mean 39.4 | DM/GDM: Included (22%) |
| **Melamed 2011** | Retrospective cohort, Single hospital, Israel | N= 4765 (431)  Risk: Mixed  Selection: All deliveries with USS within 3 days of delivery.  DM/GDM and SROM excluded. | EFW (multiple) and AC  Hadlock (AC/FL/BPD)  Hadlock (AC/FL/HC)  Hadlock (AC/FL/BPD/HC)  Hadlock (AC/FL)  Shepard (AC/BPD)  Threshold: >4000g,>36cm  Blinded: No | Within 3 days of delivery | BW >4000g | Mean 38.1 | DM/GDM: Excluded |
| **Miller 1986** | Retrospective cohort,  Single Hospital, Luisiana, USA | N= 150 (28)  Risk: Mixed  Selection: Term only, included diabetes, PET, prior CS.  Excluded SGA | EFW  Hadlock (AC/FL)  Shepard (AC/BPD)  Threshold: >4000g  Blinded: No | Within 7 days of delivery | BW >4000g | Term  (Mean ga not reported) | DM/GDM: Included |
| **Miller 1988** | Retrospective cohort,  Single Hospital, Luisiana, USA | N= 382 (58)  Risk: Mixed  Selection: term only, excluded SROM | EFW and AC  Hadlock (AC/FL/BPD)  Threshold: EFW >4100g, AC >36.4cm  Blinded: No | Within 7 days of delivery.  Mean ga 275.8 days | BW >4000g | Mean ga 279.1 days. | DM/GDM: Not reported |
| **Nahum 2003** | Retrospective cohort,  Single hospital, California, USA | N= 74 (12)  Risk: Mixed  Selection: Only included Hispanic ethnicity, term only. | EFW (11 formulas)  Hadlock (AC/FL/BPD)  Hadlock (AC/FL/HC)  Hadlock (AC/FL/BPD/HC)  Hadlock (AC/BPD)  Shepard (AC/BPD)  Threshold: >4000g  Blinded: No | Within 3 weeks of delivery | BW >4000g | Term  (Mean ga not reported) | DM/GDM: Included (23.0%) |
| **Nahum 2007** | Retrospective cohort,  Single hospital, California, USA | N= 98 (16)  Risk: Low risk  Selection: Term only, Excluded medical complications (PET, DM) | EFW  Hadlock (AC/FL/BPD)  Hadlock (AC/BPD)  Hadlock (AC/FL)  Threshold: >4000g,  Blinded: No | Within 3 weeks of delivery | BW >4000g | Term  (Mean ga not reported) | DM/GDM: Excluded |
| **Nicod 2012**  **(French)** | Retrospective cohort,  Single hospital, Switzerland | N= 708 (141)  Risk: Mixed risk  Selection: Pregnancies with USS within 7 days of delivery. | EFW  Hadlock (AC/FL/BPD/HC)  Hadlock (AC/FL)  Threshold: >4000g  Blinded: No | Within 7 days of delivery | BW >4000g | Not reported | DM/GDM: Not reported |
| **O’Reilly-Green 1997** | Retrospective cohort,  Single hospital, New York, USA | N= 445 (107)  Risk: Low  Selection: Prolonged pregnancies defined as ga >40+4. | EFW  Hadlock (AC/FL/BPD)  Threshold: >4000g, >4500g  Blinded: No | Within 3 weeks of delivery | BW >4000g  BW >4500g | GA >40+4 | DM/GDM: Excluded |
| **Pates 2007** | Retrospective cohort,  Single hospital, Texas, USA | N= 3115 (239)  Risk: Mixed  Selection: Those with clinically indicated USS within 7 days of delivery. | EFW and AFI  Hadlock (AC/FL/BPD/HC)  Threshold: >4000g, AFI >20cm (95^th^ centile)  Blinded: No | Within 7 days of delivery | BW >4000g | Not reported | DM/GDM: Included (11%) |
| **Peregrine 2007** | Prospective cohort,  Single hospital, London, UK | N= 262 (48)  Risk: Mixed  Selection: Pregnancies with ga >35+6 undergoing IOL, Excluded those withIUD or antepartum haemorrhage. | EFW  Hadlock (AC/FL)  Shepard (AC/BPD)  Threshold: >4000g  Blinded: Yes | Exactly before IOL | BW >4000g | Median ga 41 weeks. | DM/GDM: Not reported |
| **Pollack 1992** | Retrospective cohort,  Single hospital, New York, USA | N= 519 (119)  Risk: Mixed  Selection: Postdate pregnancies >41 weeks | EFW  Hadlock (AC/FL)  Threshold: >4000g, >4500g  Blinded: No | Within 7 days of delivery | BW >4000g | >41 weeks | DM/GDM: Not reported |
| **Rossavik 1993** | Retrospective cohort,  Single hospital, Oklahoma, USA | N= 498 (36)  Risk: Mixed  Selection: Infants with USS within 2 weeks of delivery (if ga >38w) or within 1 week of delivery (if ga <38w) | EFW  Hadlock (AC/FL/HC)  Threshold: >4000g  Blinded: No | Within 2 weeks of delivery (if ga >38w) or within 1 week of delivery (if ga <38w) | BW >4000g | Not reported | DM/GDM: Not reported |
| **Sapir 2017**  **(Abstract)** | Retrospective cohort  Single Hospital, Israel | N=6214  Risk: Mixed  Selection: term only, no GDM with scan within 7 days of delivery | EFW, AC  Threshold: >4000g, >4500g, AC>39cm  Blinded: No | Wiothin 1 week of delivery | Shoulder dystocia | Term (not specified) | DM/GDM: Excluded |
| **Smith 1997** | Retrospective cohort,  Single hospital, Glasgow, UK | N= 1213 (16)  Risk: Mixed  Selection: Non-diabetic pregnancies with USS within 7 days of delivery. | EFW and AC  Hadlock (AC/FL)  Threshold: >4000g, >4500g, AC >36cm, AC >38cm  Blinded: No | Within 7 days of delivery | BW >4500g | Not reported | DM/GDM: Excluded |
| **Sovio 2018** | Prospective cohort,  Single hospital, Cambridge, UK | N= 3866 (177)  Risk: Universal  Selection: Unselected n nulliparous women that delivered after 36 weeks. | EFW, ACGV  Hadlock (AC/FL/BPD/HC)  Threshold: >90^th^ centile (population/customised)  Blinded: Yes | Regular research scan at 36 weeks  (median 36.4 weeks) | BW >90^th^ centile  BW >97^th^ centile  BW >4000g, BW >4500g, shoulder dystocia, metabolic acidosis, 5-min Apgar <7, NICU admission, severe neonatal morbidity, neonatal hypoglycaemia, neonatal jauntice | Median 40.4 weeks. | DM/GDM: Included (4.3%) |
| **Sritippayawan 2007** | Prospective cohort, Single Hospital, Thailand | N= 328 (3)  Risk: Low risk  Selection: Pregnancies >34 weeks. Excluded IUFD, any medical complication. | EFW  Hadlock (AC/FL/BPD/HC)  Threshold: >4000g  Blinded: No | >34 weeks  Mean interval 16.9 days from delivery | BW >4000g | Mean ga 39.4 weeks. | DM/GDM: Excluded |
| **Sylvestre 2000** | Retrospective cohort, Single Hospital, New York, USA | N= 656 (147)  Risk: Low risk  Selection: Postdate pregnancies only (>41 weeks) | EFW (Hadlock or Shepard/Not specified)  Threshold: >4000g  Blinded: No | >41 weeks | BW >4000g | 41.3 weeks | DM/GDM: Not reported |
| **Weiner 2002** | Prospective cohort, Single centre, Israel | N= 315 (134)  Risk: Mixed risk  Selection: Offered routine clinical screening to all womenat term. Those with suspected EFW >3700g had USS. Only included those with USS with 3 days of delivery. | EFW  Shepard (AC/BPD)  Threshold: >4000g  Blinded: No | USS with 3 days of delivery. | BW >4000g  BW >4500g  Shoulder dystocia | 40.1 weeks for both groups. | DM/GDM: Included (9.2%) |

* The references are in the main body of the manuscript
