# Supplementary material for: Universal third-trimester ultrasonic screening using fetal macrosomia in the prediction of adverse perinatal outcome: A systematic review and meta-analysis of diagnostic test accuracy
Source: PLoS Med. 2020 Oct 13;17(10):e1003190. doi: 10.1371/journal.pmed.1003190 (PMC7553291; doi:10.1371/journal.pmed.1003190)
Supplement: S2 Table — (DOCX) [file pmed.1003190.s004.docx]

S2_Table. List of studies excluded from the meta-analysis and reason for exclusion

| **Study** | **Reason for exclusion** |
| --- | --- |
| **Al-Inany et al, 2001^1^** | Intrapartum only |
| **Araujo Junior et al, 2007^2^** | Non-systematic review |
| **Bamberg et al, 2013^3^** | Non-systematic review |
| **Barel et al, 2014^4^** | Not a diagnostic accuracy study |
| **Chaabane et al, 2013^5^** | Case-control study |
| **Chandrasekaran et al, 2016^6^** | High risk, only included obese patients |
| **Chauhan et al, 1995^7^** | Intrapartum only |
| **Chauhan et al, 1998^8^** | Intrapartum only |
| **Chauhan et al, 2000^9^** | No data available to generate 2x2 tables |
| **Coomarasamy et al, 2005^10^** | Systematic review including both high and low risk pregnancies |
| **Delapapa et al, 1991^11^** | High risk, only included pregnancies with suspected LGA |
| **Hart et al, 2010^12^** | High risk, only included pregnancies with birthweight >4000g |
| **Hoopmann et al, 2010^13^** | High risk, only included pregnancies with birthweight >4000g |
| **Jazayeri et al , 2001^14^** | Case-control study |
| **King et al, 2012^15^** | No data available to generate 2x2 tables |
| **Lindell et al, 2012^16^** | High risk, only included pregnancies with suspected LGA |
| **Lindell et al, 2013^17^** | No data available to generate 2x2 tables |
| **Little et al, 2012^18^** | No data available to generate 2x2 tables |
| **Mazouni et al, 2007^19^** | High risk, only included pregnancies with suspected LGA |
| **Melamed et al, 2010^20^** | Case-control study |
| **Mongelli et al, 2005^21^** | No data available to generate 2x2 tables |
| **Moore et al, 2015^22^** | High risk, only included patients with gestational diabetes. |
| **Ocer et al, 1999^23^** | Case-control study |
| **Parra Saavedra et al, 2015^24^** | Abstract - Not enough data to generate 2x2 tables |
| **Parry et al, 2000^25^** | High risk, only included pregnancies with EFW >4000g |
| **Pilalis et al, 2012^26^** | No data available to generate 2x2 tables |
| **Rotmensch et al , 1999^27^** | Intrapartum only |
| **Santolaya-Forgas, 1994^28^** | Intrapartum only |
| **Sood et al, 1995^29^** | Intrapartum only |
| **Souka et al, 2013^30^** | No data available to generate 2x2 tables |
| **Valent et al, 2017^31^** | High risk, only included pregnancies with diabetes mellitus |

**References of the studies excluded from the meta-analysis**

1. H. Al-Inany, N. Alaa, M. Momtaz and M. Abdel Badii. Intrapartum prediction of macrosomia: accuracy of abdominal circumference estimation. *Gynecologic & Obstetric Investigation* 2001; **51**: 116-119.

2. E. Araujo Junior, A. B. Peixoto, A. C. Zamarian, J. Elito Junior and G. Tonni. Macrosomia. *Best Practice & Research in Clinical Obstetrics & Gynaecology* 2017; **38**: 83-96.

3. C. Bamberg, L. Hinkson and W. Henrich. Prenatal detection and consequences of fetal macrosomia. *Fetal Diagnosis & Therapy* 2013; **33**: 143-148.

4. O. Barel, R. Maymon, Z. Vaknin, J. Tovbin and N. Smorgick. Sonographic fetal weight estimation - is there more to it than just fetal measurements? *Prenatal Diagnosis* 2014; **34**: 50-55.

5. K. Chaabane, K. Trigui, D. Louati, S. Kebaili, H. Gassara, A. Dammak, H. Amouri and M. Guermazi. Antenatal macrosomia prediction using sonographic fetal abdominal circumference in South Tunisia. *The Pan African medical journal* 2013; **14**: 111.

6. S. Chandrasekaran, J. A. Bastek, A. L. Turitz and C. P. Durnwald. A prediction score to assess the risk of delivering a large for gestational age infant among obese women. *Journal of Maternal-Fetal and Neonatal Medicine* 2016; **29**: 22-26.

7. S. P. Chauhan, B. D. Cowan, E. F. Magann, T. H. Bradford, W. E. Roberts and J. C. Morrison. Intrapartum detection of a macrosomic fetus: clinical versus 8 sonographic models. *Australian & New Zealand Journal of Obstetrics & Gynaecology* 1995; **35**: 266-270.

8. S. P. Chauhan, N. W. Hendrix, E. F. Magann, J. C. Morrison, S. P. Kenney and L. D. Devoe. Limitations of clinical and sonographic estimates of birth weight: Experience with 1034 parturients. *Obstetrics and Gynecology* 1998; **91**: 72-77.

9. S. P. Chauhan, D. J. West, J. A. Scardo, J. M. Boyd, J. Joiner and N. W. Hendrix. Antepartum detection of macrosomic fetus: clinical versus sonographic, including soft-tissue measurements. *Obstetrics & Gynecology* 2000; **95**: 639-642.

10. A. Coomarasamy, M. Connock, J. Thornton and K. S. Khan. Accuracy of ultrasound biometry in the prediction of macrosomia: a systematic quantitative review. *Bjog* 2005; **112**: 1461-1466.

11. E. H. Delpapa and E. Mueller-Heubach. Pregnancy outcome following ultrasound diagnosis of macrosomia. *Obstetrics & Gynecology* 1991; **78**: 340-343.

12. N. C. Hart, A. Hilbert, B. Meurer, M. Schrauder, M. Schmid, J. Siemer, M. Voigt and R. L. Schild. Macrosomia: a new formula for optimized fetal weight estimation.[Erratum appears in Ultrasound Obstet Gynecol. 2011 Feb;37(2):254]. *Ultrasound in Obstetrics & Gynecology* 2010; **35**: 42-47.

13. M. Hoopmann, H. Abele, N. Wagner, D. Wallwiener and K. O. Kagan. Performance of 36 different weight estimation formulae in fetuses with macrosomia. *Fetal Diagn Ther* 2010; **27**: 204-213.

14. A. Jazayeri, J. A. Heffron, R. Phillips and W. N. Spellacy. Macrosomia prediction using ultrasound fetal abdominal circumference of 35 centimeters or more. *Obstetrics & Gynecology* 1999; **93**: 523-526.

15. J. R. King, L. M. Korst, D. A. Miller and J. G. Ouzounian. Increased composite maternal and neonatal morbidity associated with ultrasonographically suspected fetal macrosomia. *Journal of Maternal-Fetal and Neonatal Medicine* 2012; **25**: 1953-1959.

16. G. Lindell, K. A. K and K. Marsal. Ultrasound weight estimation of large fetuses. *Acta Obstetricia et Gynecologica Scandinavica* 2012; **91**: 1218-1225.

17. G. Lindell, K. Marsal and K. Kallen. Predicting risk for large-for-gestational age neonates at term: a population-based Bayesian theorem study. *Ultrasound in obstetrics & gynecology : the official journal of the International Society of Ultrasound in Obstetrics and Gynecology* 2013; **41**: 398-405.

18. S. E. Little, A. G. Edlow, A. M. Thomas and N. A. Smith. Estimated fetal weight by ultrasound: a modifiable risk factor for cesarean delivery? *American Journal of Obstetrics & Gynecology* 2012; **207**: 309.e301-306.

19. C. Mazouni, R. Rouzier, R. Ledu, H. Heckenroth, B. Guidicelli and M. Gamerre. Development and internal validation of a nomogram to predict macrosomia. *Ultrasound in Obstetrics & Gynecology* 2007; **29**: 544-549.

20. N. Melamed, Y. Yogev, I. Meizner, R. Mashiach and A. Ben-Haroush. Sonographic prediction of fetal macrosomia: the consequences of false diagnosis. *J Ultrasound Med* 2010; **29**: 225-230.

21. M. Mongelli and R. Benzie. Ultrasound diagnosis of fetal macrosomia: a comparison of weight prediction models using computer simulation. *Ultrasound in Obstetrics & Gynecology* 2005; **26**: 500-503.

22. G. S. Moore, A. L. Post, N. A. West, J. E. Hart and A. M. Lynch. Fetal weight estimation in diabetic pregnancies using the gestation-adjusted projection method: comparison of two timing strategies for third-trimester sonography. *Journal of ultrasound in medicine : official journal of the American Institute of Ultrasound in Medicine* 2015; **34**: 971-975.

23. F. Ocer, S. Kaleli, E. Budak and E. Oral. Fetal weight estimation and prediction of fetal macrosomia in non-diabetic pregnant women. *European Journal of Obstetrics, Gynecology, & Reproductive Biology* 1999; **83**: 47-52.

24. M. A. Parra Saavedra, S. Triunfo, F. Crovetto, E. Gratacos and F. Figueras. Detection of late pregnancy Large-for-gestational age fetuses (LGA ) by ultrasound and doppler evaluation at routine third trimester scan in adequate-for-gestational age (AGA) pregnancies. *Journal of Perinatal Medicine Conference: 12th World Congress of Perinatal Medicine* 2015; **43**.

25. S. Parry, C. P. Severs, H. M. Sehdev, G. A. Macones, L. M. White and M. A. Morgan. Ultrasonographic prediction of fetal macrosomia. Association with cesarean delivery. *J Reprod Med* 2000; **45**: 17-22.

26. A. Pilalis, A. P. Souka, I. Papastefanou, V. Michalitsi, P. Panagopoulos, C. Chrelias and D. Kassanos. Third trimester ultrasound for the prediction of the large for gestational age fetus in low-risk population and evaluation of contingency strategies. *Prenatal Diagnosis* 2012; **32**: 846-853.

27. S. Rotmensch, C. Celentano, M. Liberati, G. Malinger, O. Sadan, U. Bellati and M. Glezerman. Screening efficacy of the subcutaneous tissue width/femur length ratio for fetal macrosomia in the non-diabetic pregnancy. *Ultrasound in Obstetrics & Gynecology* 1999; **13**: 340-344.

28. J. Santolaya-Forgas, W. J. Meyer, D. W. Gauthier and D. Kahn. Intrapartum fetal subcutaneous tissue/femur length ratio: an ultrasonographic clue to fetal macrosomia. *American Journal of Obstetrics & Gynecology* 1994; **171**: 1072-1075.

29. A. K. Sood, M. Yancey and D. Richards. Prediction of fetal macrosomia using humeral soft tissue thickness. *Obstetrics & Gynecology* 1995; **85**: 937-940.

30. A. P. Souka, I. Papastefanou, A. Pilalis, V. Michalitsi, P. Panagopoulos and D. Kassanos. Performance of the ultrasound examination in the early and late third trimester for the prediction of birth weight deviations. *Prenatal Diagnosis* 2013; **33**: 915-920.

31. A. M. Valent, T. Newman, S. Kritzer, K. Magner and C. R. Warshak. Accuracy of Sonographically Estimated Fetal Weight Near Delivery in Pregnancies Complicated With Diabetes Mellitus. *Journal of ultrasound in medicine : official journal of the American Institute of Ultrasound in Medicine* 2017; **36**: 593-599.
