# Supplementary figures and images for: Universal third-trimester ultrasonic screening using fetal macrosomia in the prediction of adverse perinatal outcome: A systematic review and meta-analysis of diagnostic test accuracy
Source: PLoS Med. 2020 Oct 13;17(10):e1003190. doi: 10.1371/journal.pmed.1003190 (PMC7553291; doi:10.1371/journal.pmed.1003190)

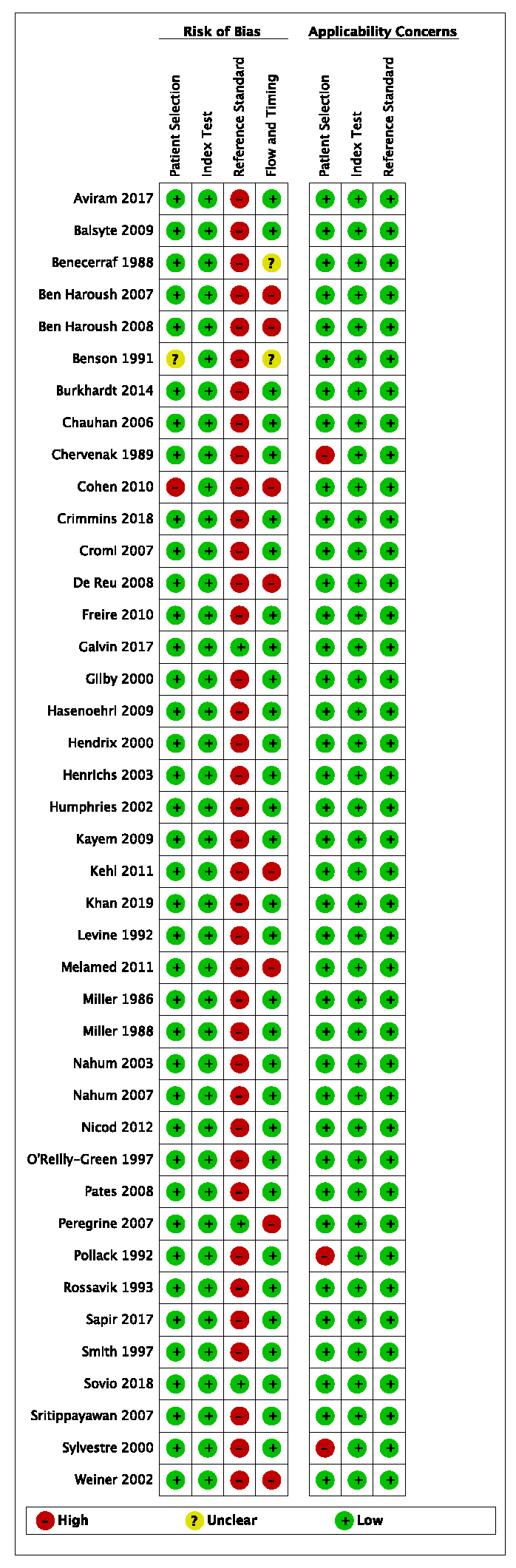

Supplement: S1 Fig — QUADAS 2, Quality Assessment of Diagnostic Accuracy Studies (TIFF) [file pmed.1003190.s005.tiff]

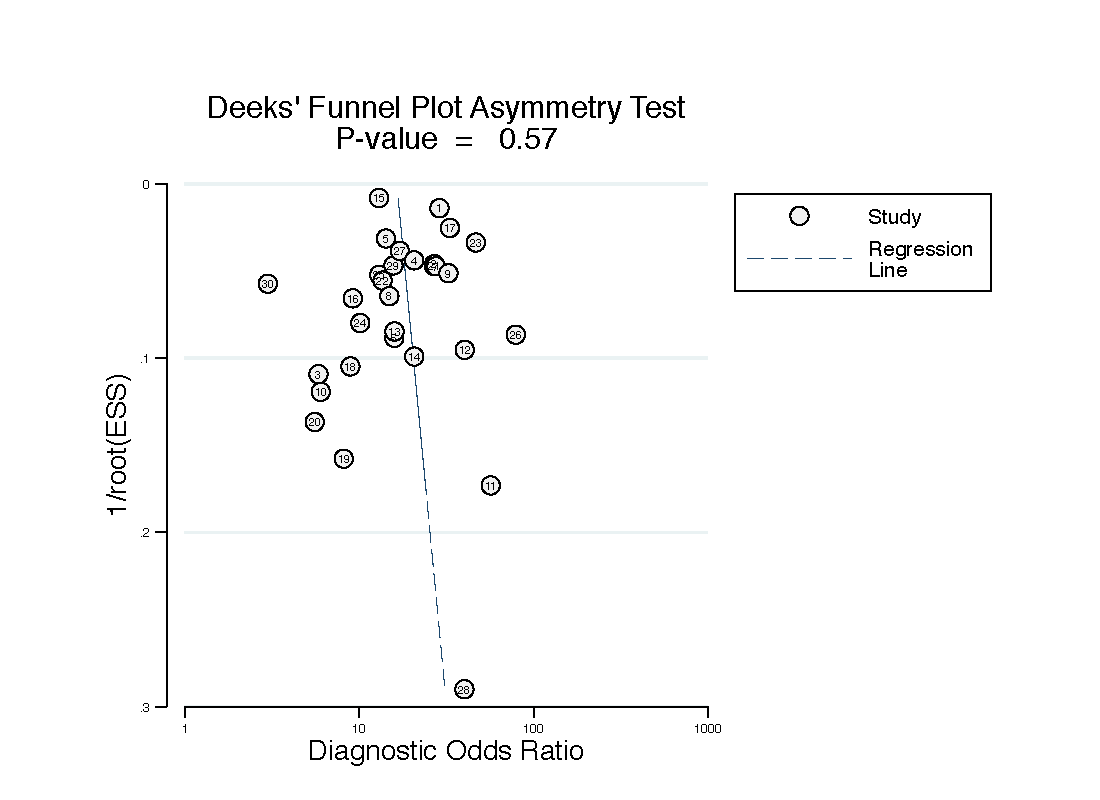

Supplement: S2 Fig — (TIFF) [file pmed.1003190.s006.tiff]
