# Supplementary material for: Universal third-trimester ultrasonic screening using fetal macrosomia in the prediction of adverse perinatal outcome: A systematic review and meta-analysis of diagnostic test accuracy
Source: PLoS Med. 2020 Oct 13;17(10):e1003190. doi: 10.1371/journal.pmed.1003190 (PMC7553291; doi:10.1371/journal.pmed.1003190)
Supplement: Attachment — Submitted filename: moraitis.pdf [file pmed.1003190.s007.pdf]

This paper describes a systematic review and meta–analysis of diagnostic tests in obstetrics.

I was asked for a statistical report and I interpret that to include all aspects of the design and conduct of the study.

Points of detail

Page 10 Some of the present authors were also involved in some of the primary studies. This is only to be expected of course but we could perhaps use a sentence here outlining what precautions were taken to avoid them quality scoring their own studies.

Page 10 I am not clear what flow and timing means. This may not matter since it may be clear to clinical readers (to demonstrate my ignorance I did not even know what macrosomia is, but I do now).

Page 11 I am more familiar with the term Reitsma model. Perhaps Reitsma could be added as a parenthesis after 'bivariate logit–normal'.

Page 12 Giving the excluded studies is very helpful.

Page 13 My initial reaction was to say: Where are the confidence intervals? Of course they are in the Table. Perhaps change to 'Full details of the summary diagnostic performance are presented in Table 1. In summary an estimated EFW . . . '

Page 16 and Figure 3 The caption could be substantially extended. What are: the square, the green solid line, the red dashed curve, the green dashed curve and what governs the size of the circles? I can guess all of these but it should be made explicit.

Page 16 and Figures 4 and 5 I know the text on page 16 states these are diagnostic odds ratios but I think the captions to the figures should too. Figure 4 needs OR changing to DOR to be compatible to Figure 5.

Summary

Mostly comments about clarification.

Michael Dewey
