# Supplementary material for: Universal third-trimester ultrasonic screening using fetal macrosomia in the prediction of adverse perinatal outcome: A systematic review and meta-analysis of diagnostic test accuracy
Source: PLoS Med. 2020 Oct 13;17(10):e1003190. doi: 10.1371/journal.pmed.1003190 (PMC7553291; doi:10.1371/journal.pmed.1003190)
Supplement: Attachment — Submitted filename: PMEDICINE-D-20-0008R1- response to reviewers.docx [file pmed.1003190.s008.docx]

Requests from the editors:


Title – Please insert a colon before the study descriptor in Universal third trimester ultrasonic screening using fetal macrosomia in the prediction of adverse perinatal outcome, a systematic review and meta-analysis of diagnostic test accuracy.
Abstract – please use p values, where possible, where 95%Cis are used (also elsewhere, as needed) and please add a sentence on the study’s limitations as the final sentence of the abstract
Thank you for providing the PRISMA checklist – please use sections and paragraphs instead of page numbers as these can change on formatting and revision.

**R1: We have made the above changes. Regarding the p-values, they are not typically reported in the estimation of sensitivities or likelihood ratios as the aim is to quantify the diagnostic performance rather than test a null hypothesis.**

Comments from the reviewers:

Reviewer #1: The study question (3rd trimester US screening for macrosomia) is a well-known, but not evidence based answered one. It is an important problem to the community of clinical obstetricians in the high- and middle-income countries.
The design and the methodology of the study is well organized. The results are clearly written and the consequnezes for the day-to-day work of obstetrical clinicians and for future scientific study questions are mentioned.
The manuscript can be published without revision, may I ask for correction of some typing errors (like in the short running title marcosomia - macrosomia!).

**R2: Done**


Reviewer #2:

This paper describes a systematic review and meta{analysis of diagnostic

tests in obstetrics.

I was asked for a statistical report and I interpret that to include all aspects

of the design and conduct of the study.

Points of detail

Page 10 Some of the present authors were also involved in some of the

primary studies. This is only to be expected of course but we could

perhaps use a sentence here outlining what precautions were taken to

avoid them quality scoring their own studies.

**R3: We used exactly the same methods when analysing and assessing the quality of the studies we were involved in. We have added a sentence to explain that.**

Page 10 I am not clear what flow and timing means. This may not matter

since it may be clear to clinical readers (to demonstrate my ignorance

I did not even know what macrosomia is, but I do now).

**R4: We used the term “flow and timing” as suggested by the QUADAS-2 tool. We have added a sentence to clarify its meaning in this specific setting.**

Page 11 I am more familiar with the term Reitsma model. Perhaps Reitsma

could be added as a parenthesis after `bivariate logit{normal'.

**R5: Done**

Page 12 Giving the excluded studies is very helpful.

Page 13 My initial reaction was to say: Where are the confidence intervals?

Of course they are in the Table. Perhaps change to `Full details of the

summary diagnostic performance are presented in Table 1. In summary

an estimated EFW . . . '

**R6: Done**

Page 16 and Figure 3 The caption could be substantially extended. What

are: the square, the green solid line, the red dashed curve, the green

dashed curve and what governs the size of the circles? I can guess all

of these but it should be made explicit.

**R7: Done**

Page 16 and Figures 4 and 5 I know the text on page 16 states these are

diagnostic odds ratios but I think the captions to the figures should too.

Figure 4 needs OR changing to DOR to be compatible to Figure 5.

**R8: Done**

Summary

Mostly comments about clarification.

Michael Dewey


Reviewer #3: Overall: a well written paper: clear and concise, easy to read, clinically relevant.
Abstract: In the abstract there is a reference to ultrasound screening and breech. Why, this is not incorporated in the introduction, nor is a reference given. Seems a bit out of place.

**R9: Sentence removed**

The aim is clear and well defined. Moreover, the authors address an important clinical problem: can shoulder dystocia be predicted?.
I have only one concern. I miss information on the inclusion of just abstracts (i.e. non-peer reviewed studies) in this systematic review and meta-analysis (since the abstract of Galvin 2017 is included) . Did the authors check if study protocols were published in a registry or were authors contacted in order to assess the risk of bias? If authors are unable to share their protocol, their study should not included. Please add.

**R10: The abstract published by Galvin et al is part of the large GENESIS study in Ireland. We have used another publication from the same cohort (which explains in detail the protocol) to assess the quality of the study. We have clarified this in the results and added the relevant reference.**
